# Research on animal models of chronic dacryocystitis in dogs

**DOI:** 10.1186/s12886-026-04974-8

**Published:** 2026-07-07

**Authors:** Xiaoye Wang, Huan Jiang, Xinyu Chen, Yu Jiang, Shun Zhan, Jianpin He, Taixiang Liu, Bing Jiang

**Affiliations:** 1 Department of Ophthalmology, Affiliated Hospital of Zunyi Medical University, Zunyi, China; 2 Department of Urology, The Third People’s Hospital of Guizhou Province, Guiyang, China

**Keywords:** Nasal lacrimal duct, Chronic dacryocystitis, Animal model, Dog

## Abstract

**Objective:**

To explore an ideal animal model of chronic dacryocystitis.

**Methods:**

(1) The anatomical structure of the lacrimal drainage system in dogs was observed; (2) The canine lacrimal drainage system was obstructed by cauterization of the nasolacrimal duct; (3) A canine model of dacryocystitis was established by combining nasolacrimal duct cauterization with inoculation of Staphylococcus aureus.

**Results:**

Compared with inoculation of Staphylococcus aureus one week after nasolacrimal duct cauterization, the combined method of nasolacrimal duct cauterization and immediate inoculation of Staphylococcus aureus induced clinical manifestations of dacryocystitis more rapidly in dogs. The modeling success rate was 83.3%.

**Conclusion:**

Our experimental data demonstrate that the anatomical structure of the canine lacrimal duct is similar to that of humans, providing a solid anatomical foundation for modeling chronic dacryocystitis. The method of nasolacrimal duct cauterization combined with Staphylococcus aureus injection can rapidly and effectively induce canine dacryocystitis manifestations while being simple and feasible to perform. Therefore, dogs can be considered as a suitable animal model for further research on chronic dacryocystitis. This model reliably reproduces key pathological features—including chronic inflammation, ductal obstruction, and bacterial persistence—enabling mechanistic studies and therapeutic evaluation. Its reproducibility and clinical relevance support translational applications in developing novel interventions for human chronic dacryocystitis. If a more persistent chronic inflammatory model is to be constructed, further optimization of surgical parameters and inoculation protocols will still be required.

## Introduction

Chronic dacryocystitis is a common infectious disease of the lacrimal drainage system in ophthalmology [1]. It is usually associated with nasolacrimal duct obstruction and is characterized by epiphora and increased discharge [2]. This condition may lead to serious ocular complications, such as acute dacryocystitis, keratitis, and orbital cellulitis [3, 4], and it is also considered a contraindication for common intraocular surgeries, including glaucoma and cataract surgery [5]. However, the pathogenesis of chronic dacryocystitis remains unclear. Establishing appropriate animal models of this disease is therefore essential to facilitate research on its pathogenesis, progression, treatment, and prognosis. To date, rabbits have been the most commonly used animal model for chronic dacryocystitis and nasolacrimal duct obstruction [6]. Nevertheless, their representativeness remains controversial, as significant differences exist between rabbits and humans in terms of gross anatomy and histological structure of the lacrimal drainage system. In particular, rabbits lack goblet cells and a lacrimal sac structure comparable to those found in humans [7, 8]. Therefore, considering anatomical characteristics, accessibility, and sample size, our research group proposes the use of medium-sized Chinese native dogs as an experimental animal model for the study of chronic dacryocystitis.

## Materials and methods

### Experimental animals

A total of 30 Chinese native dogs without ocular diseases (such as dacryocystitis, conjunctivitis, eyelid disorders, or globe diseases) or craniofacial diseases were included in this study. The animals were of either sex and weighed approximately 10–12 kg.All animals were provided by the Animal Experimental Center of Zunyi Medical University. All experimental procedures were approved by the Animal Ethics Committee of Zunyi Medical University (Ethics approval No. ZMU21-2301-009).

### Experimental equipment and reagents

An ophthalmic operating microscope, a high-speed electric drill, a high-frequency electrosurgical unit, xylazine hydrochloride injection, iohexol injection (300 mg I/mL), and a Staphylococcus aureus suspension (0.49–0.63 × 10⁸ CFU/mL) were used in this study.

### Experimental methods

#### Anatomical characteristics of the canine lacrimal drainage system

**Fig. 1:**
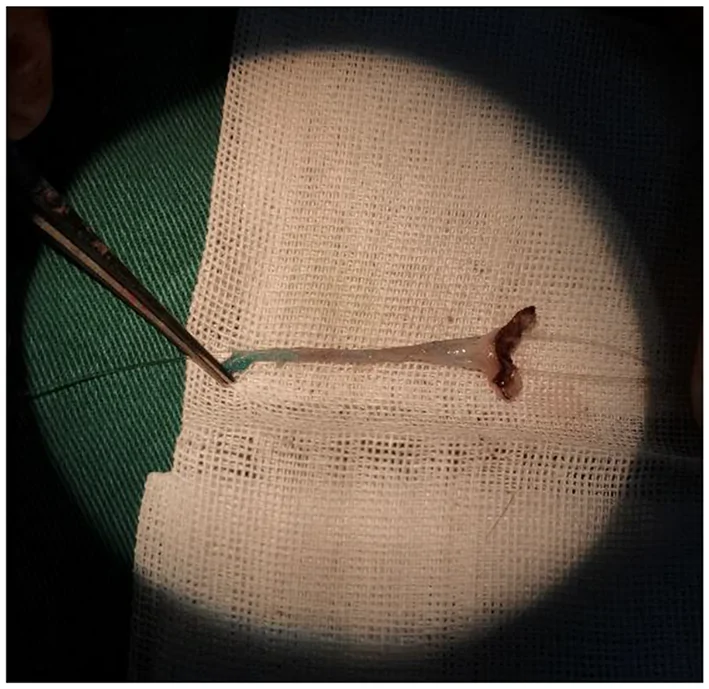
Schematic diagram of the complete lacrimal duct structure in dogs

Three Chinese rural dogs were enrolled in this study. Xylazine hydrochloride injection (0.1 mL/kg) was administered intramuscularly into the hindlimb muscles. After the anesthesia took effect, the subcutaneous and muscular tissues in the lacrimal sac area were dissected to expose the lacrimal sac and nasolacrimal duct, and the entire lacrimal passage was excised for observation (Fig. 1). The results showed that the dogs had upper and lower lacrimal puncta, upper and lower lacrimal canaliculi, lacrimal sacs, and nasolacrimal ducts, with no obvious common lacrimal duct.

The lengths and various parameters of the bilateral lacrimal passages of the three dogs were measured, and the measurement results were statistically analyzed using SPSS software (IBM Corp., Armonk, NY, USA; Version 29.0). All data were expressed as mean ± standard deviation (X ± S). Specifically, the length of the upper lacrimal canaliculus was 6.00 ± 0.60 mm, the length of the lower lacrimal canaliculus was 5.70 ± 0.50 mm, the area of the lacrimal sac was 15.12 ± 2.71 mm², the length of the membranous nasolacrimal duct was 22.70 ± 1.20 mm, and the outer diameter of the membranous nasolacrimal duct was 1.70 ± 0.50 mm.

**Fig. 2:**
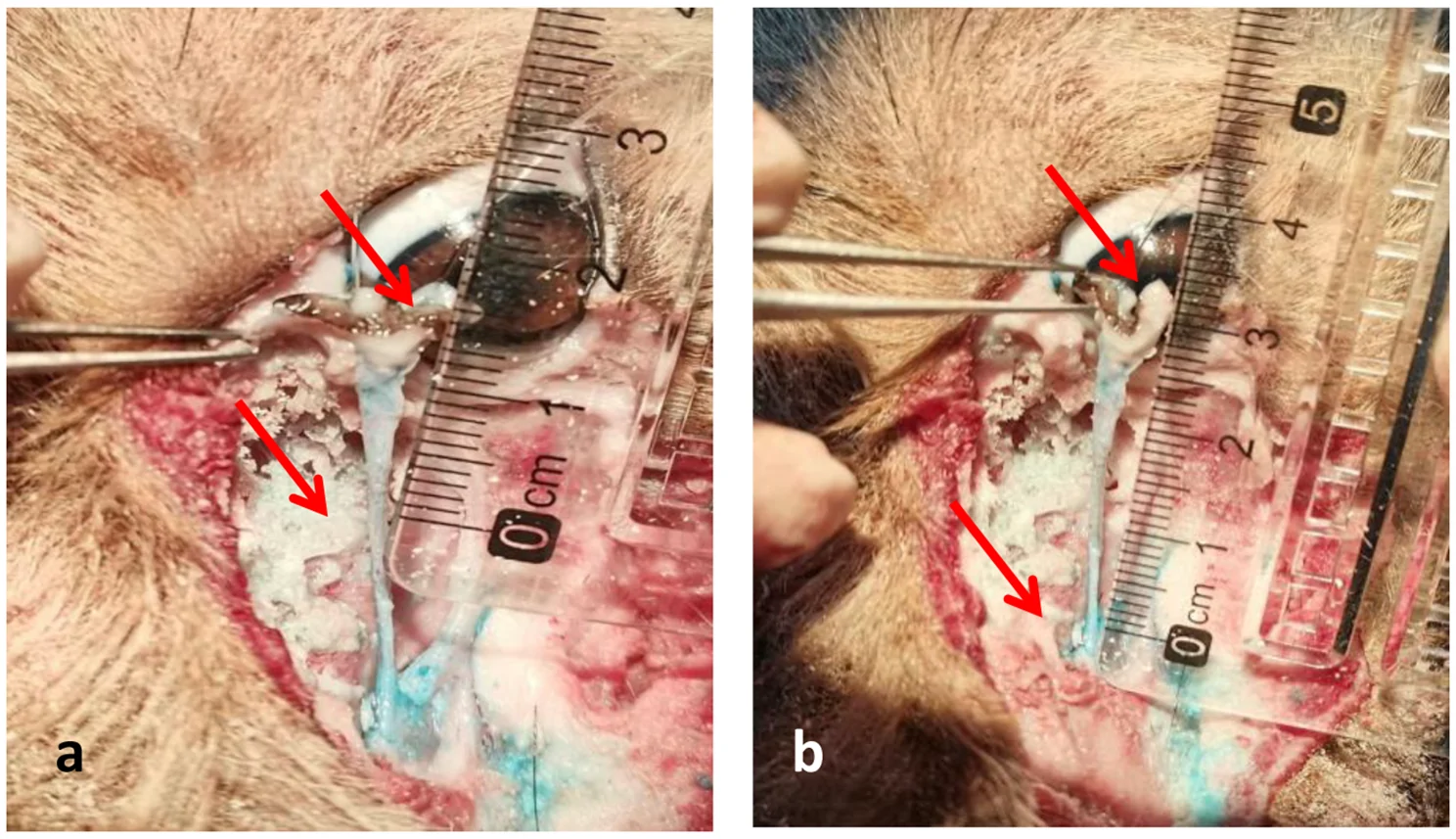
The approximate distance from the superior lacrimal punctum to the lacrimal sac in the dog indicated by arrow **a**; the approximate distance from the superior lacrimal punctum to the lower segment of the nasolacrimal duct (near the nasal cavity) in the dog indicated by arrow **b**

The distance from the lower lacrimal punctum to the lacrimal sac was 10.00 ± 1.40 mm, and the distance from the upper lacrimal punctum to the lacrimal sac was 10.60 ± 1.20 mm. Additionally, the distance from the lower lacrimal punctum to the nasal opening of the membranous nasolacrimal duct was 33.80 ± 1.20 mm, and the distance from the upper lacrimal punctum to the nasal opening of the membranous nasolacrimal duct was 33.20 ± 1.20 mm (Fig. 2a and b are schematic diagrams illustrating the measured lengths during the anatomical process).

#### Method for nasolacrimal duct obstruction in dogs

**Fig. 3:**
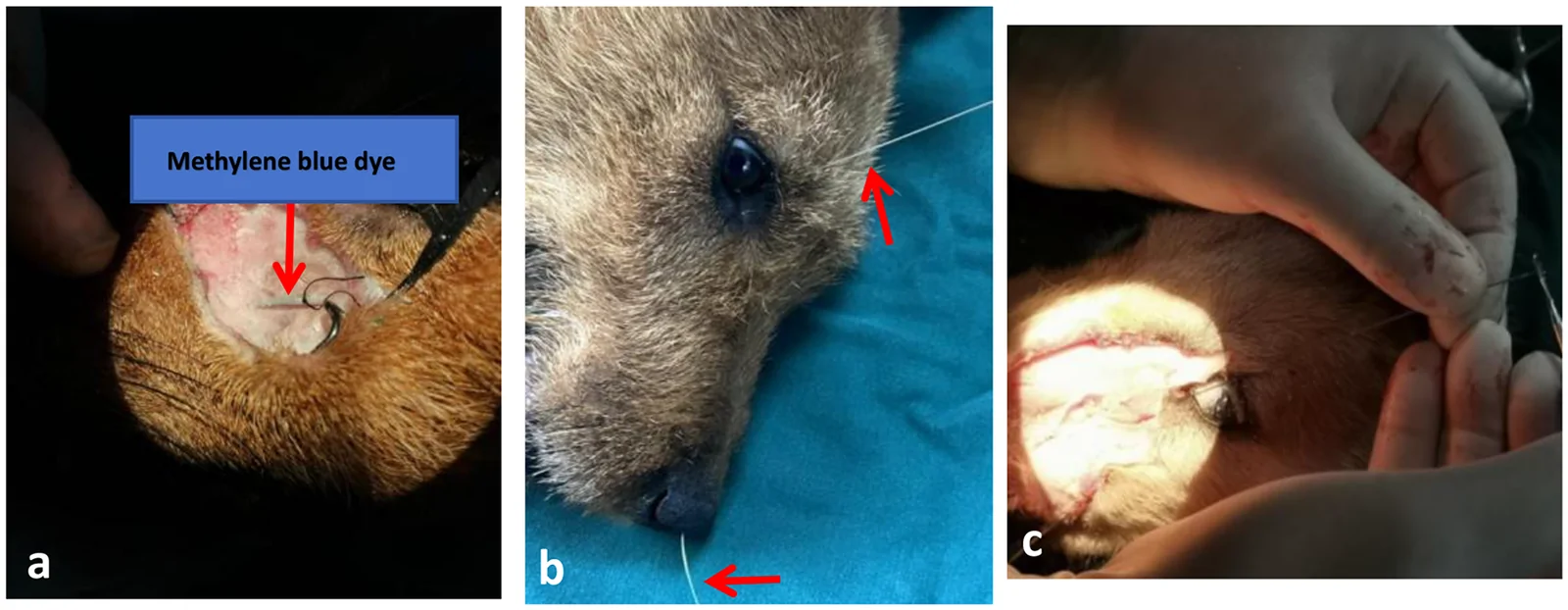
**a**: Ligation of the nasolacrimal duct is shown. After injection of methylene blue dye, the area above the ligation plane is stained, indicating the success of this method. **b**: The red arrow indicates the fishing line inserted from the superior lacrimal punctum into the nasal cavity. **c**: An actual operation image of nasolacrimal duct cauterization after power connection is shown

**Fig. 4:**
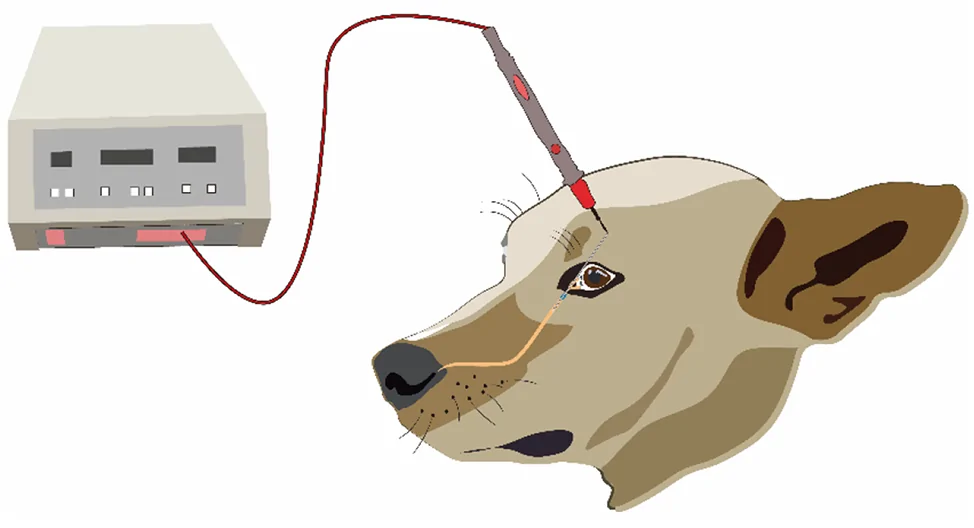
Schematic diagram of nasolacrimal duct obstruction in dogs using cauterization

**Fig. 5:**
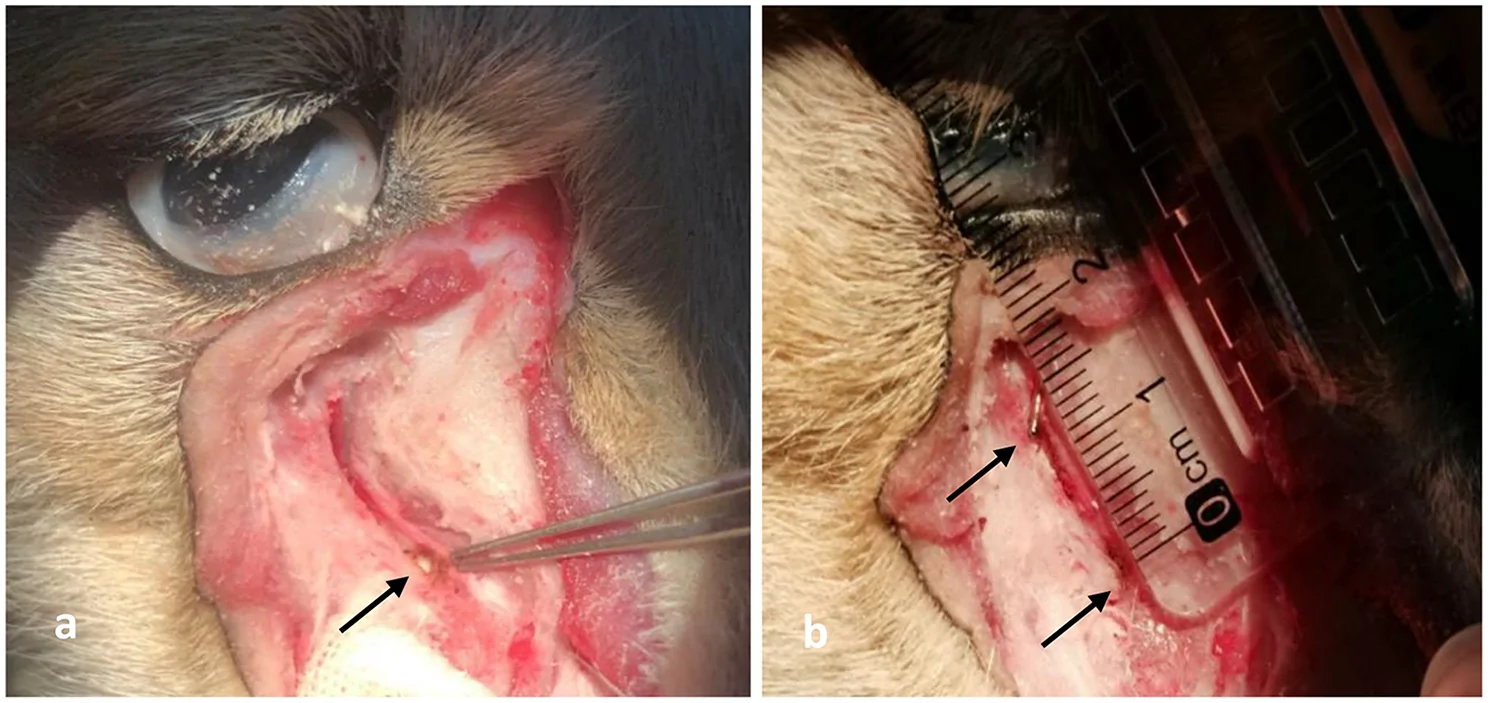
**a**: Shows the completion of cauterization and subsequent dissection of the lacrimal duct. The arrows indicate the cauterized site and the extent of cauterization on the nasolacrimal duct. **b**: The arrows indicate the safe range of the cauterizable area on the nasolacrimal duct, approximately 12 mm

Six dogs were randomly divided into 3 groups, with 2 dogs in each group (each dog had 2 eyes, totaling 4 eyes per group). The three groups were treated with different nasolacrimal duct obstruction methods respectively: Ligation method for nasolacrimal duct obstruction (Fig. 3a): The membranous nasolacrimal duct was identified through anatomical dissection, then ligated, and methylene blue dye was injected to confirm the obstruction effect. Nasal foreign body method for nasolacrimal duct obstruction (Fig. 3b): An ophthalmic fishing line was inserted through the upper lacrimal punctum, passed through the nasolacrimal duct, and exited through the nasal cavity; the foreign body was pulled upward along the fishing line to obstruct the nasolacrimal duct. Cauterization method for nasolacrimal duct obstruction (Fig. 3c): A power supply was connected, and a cautery probe was used to cauterize the nasolacrimal duct to achieve obstruction. The effect of lacrimal passage obstruction was observed. The cauterization method for nasolacrimal duct obstruction had the advantages of short time consumption, convenient operation, and easy implementation, which could prepare the lacrimal duct obstruction model more quickly. Therefore, the cauterization method was selected for nasolacrimal duct obstruction (Fig. 4 shows the practical simulation diagram). A lacrimal probe (approximately 117 mm in length) with an insulating silicone tube (exposing about 2 mm at both ends) was used, and its metal olive tip (0.5 mm in diameter) was inserted from the upper lacrimal punctum. After confirming that the metal olive tip was located in the nasolacrimal duct, the external exposed length of the lacrimal probe was measured to be about 85 mm, indicating that the probe had been inserted into the nasolacrimal duct for about 33 mm from the lacrimal punctum, which was the position of the lower segment of the nasolacrimal duct close to the nasal opening. The verification method was as follows: the fully anesthetized dog was placed on the electrode plate, and the electrode plate was connected to the high-frequency electrosurgical unit. After the power was turned on, cauterization was performed. After the cauterization of different points was completed, a high-speed electric drill was used to perform gross anatomy through the skin, muscle and bone to find the nasolacrimal duct, observe the cauterization degree and scope, and the cauterized nasolacrimal duct showed a charred appearance (marked in Fig. 5a). The safe range of the cauterized site of the nasolacrimal duct was measured to be about 12 mm (Fig. 5b), and the cauterized site was confirmed immediately. In addition, 3 dogs (6 eyes) were randomly selected to explore the cauterization parameters. The dogs were placed on the electrode plate, connected to the high-frequency electrosurgical unit, and the cauterization power was adjusted in the range of 70 W to 110 W, with a cauterization time of 1–3 s. The convulsive reaction of the dogs and the effect of lacrimal irrigation were observed. Finally, the optimal cauterization parameters were determined as follows: power of 90 W, two different points of the nasolacrimal duct were cauterized, each for 2 s. During the experiment, the dogs were anesthetized smoothly and woke up successfully after the experiment.

#### Establishment of a canine model of chronic dacryocystitis

1. **Grouping and model establishment**

Eighteen dogs were randomly divided into three groups: groups A, B, and C (groups A and B as experimental groups and group C as the control group), with six dogs in each group. All procedures were performed on the right eye.The grouping was as follows:

Group A: Inoculation of Staphylococcus aureus (0.5 mL, 0.49–0.63 × 10⁸ CFU/mL) one week after nasolacrimal duct cauterization;

Group B: Immediate inoculation of Staphylococcus aureus (0.5 mL, 0.49–0.63 × 10⁸ CFU/mL) following nasolacrimal duct cauterization;

Group C: Inoculation of Staphylococcus aureus alone (0.5 mL, 0.49–0.63 × 10⁸ CFU/mL).

2) **Periocular observation**

At 1 week, and at 1, 2, 3, and 4 months after model establishment, the right eyes of dogs in all three groups were examined for epiphora, discharge, ocular surface condition, and hair changes in the lacrimal sac region. Lacrimal irrigation was performed at 1 week and 4 months postoperatively.

3) **Imaging Examination**

Four months postoperatively, iohexol contrast medium was injected into the lacrimal sac for computed tomography (CT) examination, and normal saline was infused for magnetic resonance imaging (MRI) examination to observe the location of lacrimal duct obstruction.

4) **Pathological Examination**

Four months postoperatively, the normal lacrimal sac and the lacrimal sac with chronic dacryocystitis were collected for HE pathological examination.

## Results

Criteria for Successful Establishment of Chronic Dacryocystitis Model: The criteria for successful establishment of the chronic dacryocystitis model were as follows: epiphora accompanied by mucoid or purulent discharge, and obstruction of lacrimal irrigation with discharge reflux.

### Modeling results

#### Control group

The six dogs had no epiphora and showed unobstructed lacrimal irrigation with no discharge.

#### Group with staphylococcus aureus injection 1 week after cauterization

After model establishment, four dogs began to show epiphora symptoms approximately 1 week later; epiphora was obvious at 1 month, accompanied by matted hair, a small amount of discharge, and distinct tear stains. For lacrimal irrigation at 4 months: normal saline flowed out from the nose in 2 dogs with no reflux; normal saline refluxed upward and downward in 4 dogs, with no saline outflow from the nose and no discharge in all cases.

#### Group with immediate staphylococcus aureus injection after cauterization

**Fig. 6:**
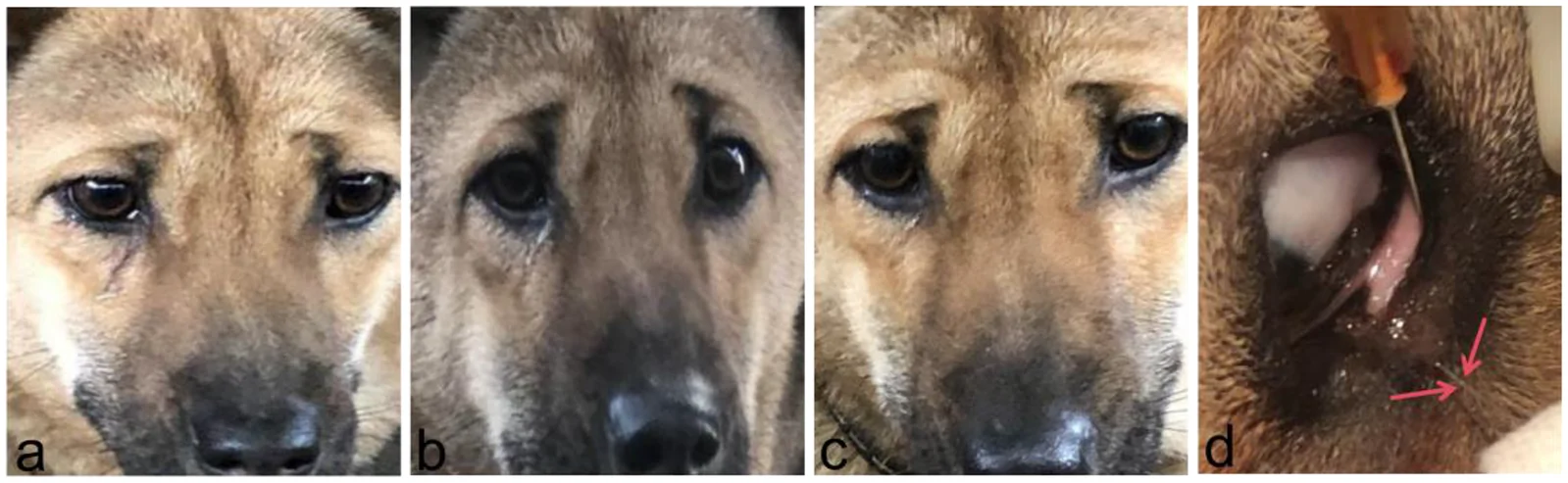
Gross findings and lacrimal irrigation at different time points after modeling in dogs. Note: (**a**) At ~1 week, marked epiphora with wet hair. (**b**) At ~1 month, mucopurulent discharge present. (**c**) At ~2 months, epiphora with discharge at the medial eyelid margin. (**d**) At~4 month, during lacrimal irrigation, mucopurulent reflux from the punctum; stringy viscous discharge after wiping (red arrow)

After model establishment, 5 dogs showed obvious lacrimation symptoms at 1 week, with moist hair and tear stains (Fig. 6a); at 1 month, obvious epiphora was present, accompanied by purulent discharge (Fig. 6b). At 2 months, tear stains and discharge were still observed (Fig. 6c). After 3 months, local lacrimation and discharge gradually alleviated. For lacrimal irrigation at 4 months: in 5 dogs, normal saline refluxed upward and downward with mucoid-purulent discharge, no saline flowed out from the nose, and no nasal outflow of saline was observed (Fig. 6d); in 1 dog, part of the saline flowed out from the lacrimal punctum and part from the nose during irrigation.

### Imaging examination

#### CT and MRI findings

**Fig. 7:**
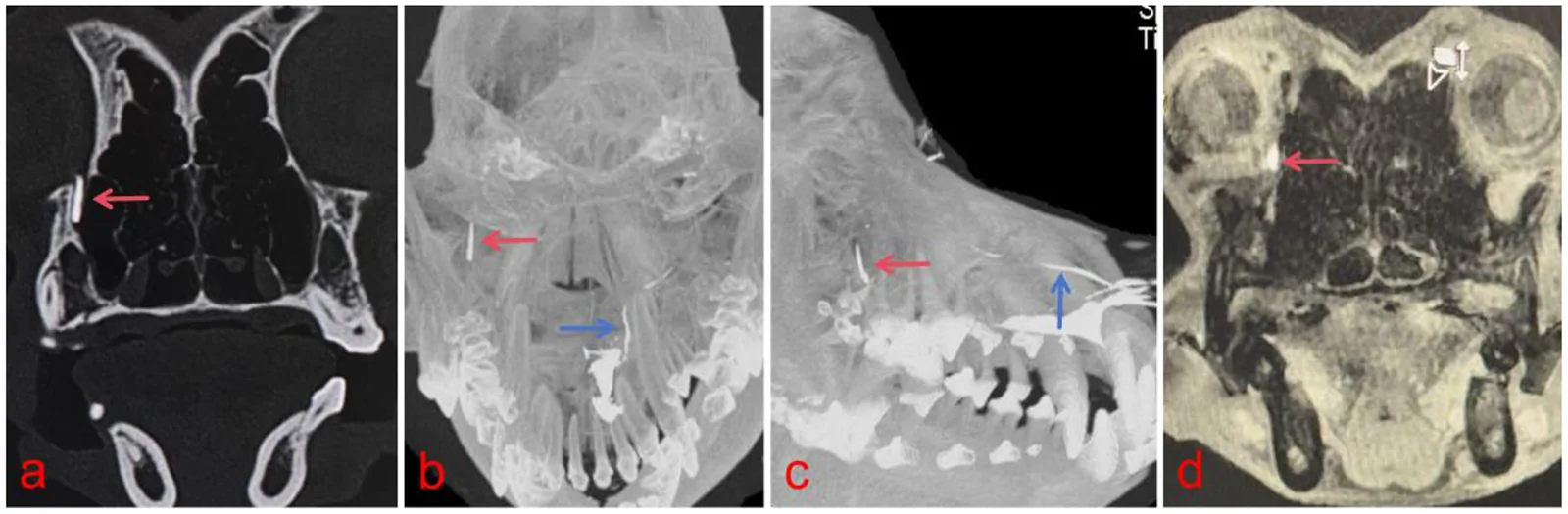
Schematic diagram of contrast-enhanced CT, CT-3D imaging, and MRI hydrography of the lacrimal duct in a dog with chronic dacryocystitis. Note: (**a**) CT of the lacrimal duct in a dog with chronic dacryocystitis: the red arrow indicates high-density contrast proximal to the obstruction. (**b**, **c**) CT 3D reconstruction: the red arrow shows contrast above the right nasolacrimal duct obstruction, and the blue arrow shows contrast reaching the nasal cavity on the left. (**d**) MRI of the lacrimal duct: the red arrow indicates high-signal intensity proximal to the obstruction

CT Examination of the Lacrimal Sac in Dogs with Chronic Dacryocystitis: The upper segment of the nasolacrimal duct was visualized, while the lower segment was not visualized, indicating nasolacrimal duct obstruction (Fig. 7a); CT Three-Dimensional (3D) Imaging: Contrast medium filling was observed above the obstruction site of the right eye nasolacrimal duct of the dog, while no contrast medium was observed in the nasal cavity, indicating obstruction of the right eye nasolacrimal duct (indicated by the red arrows in Fig. 7b and c); the visualization of the left nasolacrimal duct showed complete patency of the nasolacrimal duct, and the contrast medium could reach the nasal cavity, indicating no obstruction of the left nasolacrimal duct (indicated by the blue arrows in Fig. 7b and c). Magnetic Resonance (MR) Dacryocystography: The upper segment of the nasolacrimal duct showed high-density visualization, while the lower segment was not visualized, indicating nasolacrimal duct obstruction (Fig. 7d).

### Pathological findings

#### Normal lacrimal sac

**Fig. 8:**
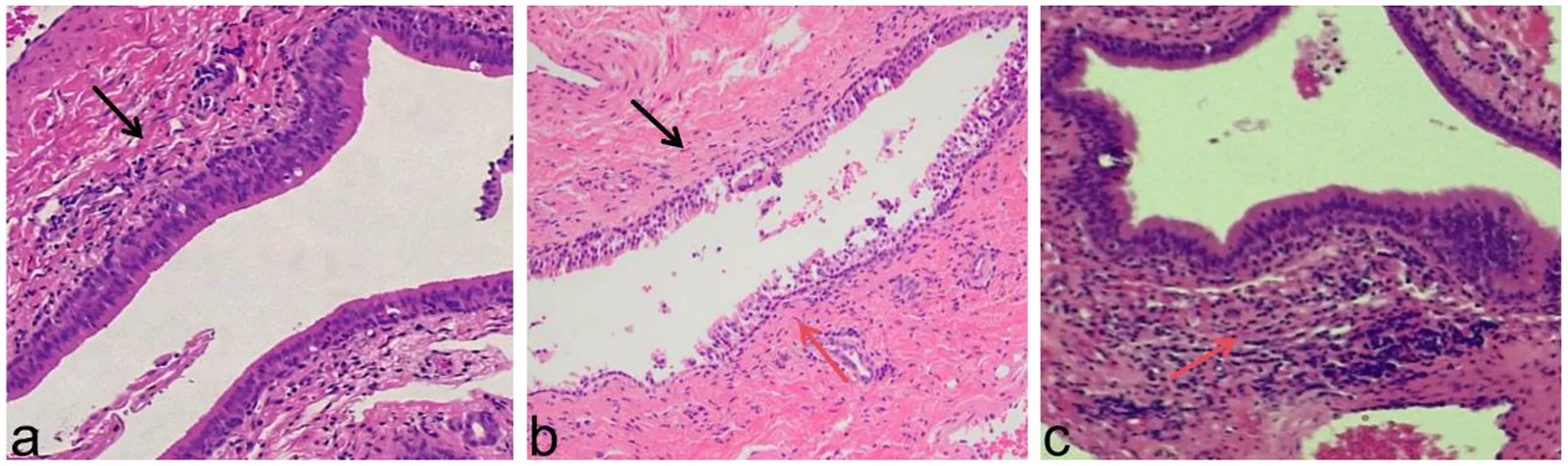
H&E staining of the normal canine lacrimal sac and chronic dacryocystitis. Note: (**a**) (as indicated by the black arrow)Normal canine lacrimal sac lined by two layers of columnar epithelium. (**b**) In dacryocystitis, epithelial necrosis and desquamation of the lacrimal sac mucosa, (the red arrow indicates) the chronic dacryocystitis group exhibits varying degrees of epithelial cell loss, from detachment to necrosis to shedding of the epithelium, while (as indicated by the black arrow) some areas show epithelial hyperplasia with 3 to 6 layers of epithelial cells visible.(**c**) In dacryocystitis, inflammatory cell infiltration in the submucosa, (the red arrow indicates) infiltration of inflammatory cells. (All images: H&E staining, light microscopy, ×10 × 10.)

The lacrimal sac was mainly composed of two layers of columnar epithelium arranged in an orderly manner. Under the epithelial basement membrane, there was loosely arranged connective tissue containing elastic fibers and other structures (the area indicated by the black arrow in Fig. 8a).

#### Lacrimal sac with chronic dacryocystitis

There were submucosal inflammatory reactions, fibrosis, and epithelial changes. There were varying degrees of epithelial cell loss, necrosis, and desquamation (the areas indicated by the black and red arrows in Fig. 8b). The mucosa of the lacrimal sac showed fibrosis, with submucosal fibrous connective tissue hyperplasia, disordered arrangement, and accompanied by chronic inflammatory cell infiltration (the area indicated by the red arrow in Fig. 8c), such as lymphocytes, macrophages, and plasma cells.

## Discussion

According to statistics by NJ Frame et al. [9], rats and mice are commonly used ophthalmic experimental animals because of their small size and low cost. However, due to marked anatomical differences between rodent and human nasolacrimal duct (NLD) systems, they are less frequently used for lacrimal duct modeling. Current studies indicate that animals with lacrimal anatomy most similar to humans include dogs, pigs, sheep, horses, and alpacas [10]. A major difference is the absence of a common canaliculus, which is typically present only in humans and primates; however, primates are difficult to obtain and costly.Although pigs are considered suitable models for craniofacial and orbital research, their lacrimal system and diseases remain unclear [11, 12], and differences in goblet cells and intraepithelial mucous glands limit their applicability. Rabbits have an incidence of chronic dacryocystitis of about 3.5% [13] and are currently the most widely used model [6, 8, 14]. Nevertheless, rabbits differ anatomically and histologically from humans, lacking goblet cells, a true lacrimal sac, and having only a single punctum and canaliculus [15–17].Hirt R et al. reported that dogs show close similarity to humans in both gross and microscopic lacrimal anatomy [18]. Our observations of Chinese native dogs confirmed the presence of superior canaliculi, a lacrimal sac, and a nasolacrimal duct, with the main difference being a longer and narrower duct, which may predispose dogs to obstruction. These features support the use of dogs as a chronic dacryocystitis model. During dissection, we also measured lacrimal duct lengths and found the distance from the superior punctum to the membranous NLD opening to be longer than that from the inferior punctum, possibly due to measurements taken along the intact duct rather than following its natural curvature.

Analyses of rabbit models of chronic dacryocystitis show various obstruction methods [6, 8, 14, 19], including simple injection of sclerosing agents to induce nasolacrimal duct obstruction, or induction of allergic obstruction followed by bacterial inoculation, with a modeling success rate of about 55%. L. Ye et al. [20] established obstruction by mechanical abrasion combined with sclerosing agent injection, while Pornpanich K et al. [21] created obstruction by scratching the lacrimal duct with a hooked probe.Because rabbit lacrimal anatomy differs markedly from that of humans and is difficult to localize, these methods may produce suboptimal obstruction. Although chronic dacryocystitis models can be achieved, clear limitations remain, indicating that even with bacterial infection, incomplete duct obstruction reduces the incidence of chronic dacryocystitis.

Therefore, our group explored two alternative methods for nasolacrimal duct (NLD) obstruction. One was NLD ligation, which provides precise obstruction but causes greater skin and muscle damage, is anatomically challenging, technically complex, and requires longer anesthesia and suturing times. Case reports indicate that foreign bodies can cause dacryocystitis in dogs [22]; thus, we considered intranasal NLD obstruction using a foreign body. A nylon thread was passed from the punctum and retrieved through the nasal cavity to occlude the duct, but high resistance and poor positional control made this approach impractical.We then used a lacrimal probe with a metal olive tip exposed ~ 2 mm at both ends and insulated by a silicone sleeve, allowing accurate localization of the tip within the membranous NLD. Insulation ensured cauterization was confined to the NLD mucosa, sparing the punctum, canaliculi, and lacrimal sac. Accordingly, NLD cauterization was selected as a simple, rapid, and safe method that shortened anesthesia time.After model establishment, lacrimal irrigation showed complete obstruction in 9 experimental eyes (Group A, *n* = 4; Group B, *n* = 5) in both the 1-week post-cauterization and immediate post-cauterization Staphylococcus aureus injection groups. This indicates rapid mucosal congestion and edema, producing temporary obstruction or stenosis, and confirms successful model creation. However, prior measurements showed the membranous NLD diameter was ~ 1 mm larger than the probe’s olive tip, suggesting that circumferential cauterization may be incomplete and that residual patency could remain in some ducts.

Paulsen FP et al. [23] suggested that inflammation or infection of the nose or eye can cause congestion and edema of the lacrimal duct mucosa, leading to temporary obstruction; recurrent inflammation may result in permanent blockage. Chronic dacryocystitis has been reported as a secondary microbial infection following nasolacrimal duct obstruction or stenosis [21]. Chen L et al. [24] found that in China the microbial culture rate in dacryocystitis is relatively low, but Gram-positive bacteria are increasingly predominant. Kathleen M et al. [25] reported abundant bacteria, including Staphylococcus spp., in rabbit dacryocystitis cultures, and Richard J et al. [26] identified Staphylococcus aureus as the most common pathogen in chronic dacryocystitis. Accordingly, S. aureus has been used in previous animal models [27].To improve model success, complete duct obstruction and accurate localization must be combined with bacterial involvement. Jin Jing et al. [27] used 0.3 ml of S. aureus suspension (1 × 10⁷ cells/ml). Considering the larger body size and lacrimal duct volume of dogs, we used 0.5 ml of S. aureus suspension at 0.49–0.63 × 10⁸ CFU/ml. From a general observation perspective, the dogs began to show lacrimation symptoms approximately 1 week after the lacrimal duct cauterization, with gradual appearance of secretions, which became more obvious around 1 month postoperatively. The lacrimation and secretions gradually alleviated at 2–3 months postoperatively, but secretion reflux was still observed during lacrimal irrigation at 4 months postoperatively. We analyze that this may be due to the enhanced disease resistance of the dogs themselves and the certain repair ability of the lacrimal duct mucosa at this stage; it may also be because the bacterial concentration adopted in the current model is insufficient, or multiple bacterial inoculations are required, thus failing to reliably maintain the long-term stability of chronic inflammation. In addition, the gradual relief of clinical symptoms may be related to the partial recanalization of the nasolacrimal duct, fibrous tissue remodeling, or the self-limiting nature of the induced inflammatory response over time. Eventually, the dogs in the experimental group showed local lacrimation accompanied by secretions, with upward and downward reflux of lacrimal irrigation and secretions, and no fluid flowed from the nasal cavity; the dogs in the control group had no lacrimation or secretions, and the lacrimal irrigation was unobstructed. This further confirms the success of our model establishment. Therefore, we believe that cauterization of the nasolacrimal duct combined with injection of Staphylococcus aureus can effectively induce dacryocystitis in dogs.

Regarding the pathogenesis of chronic dacryocystitis, previous studies suggest that loss of functional lacrimal duct structures—such as epithelial desquamation, necrosis, defects, fibrosis of the lamina propria, and vascular constriction or damage—can impair tear drainage and promote disease development [14, 23, 28]. Pathologically, Koturovic et al. [29] analyzed lacrimal sac biopsies from over 3,000 patients undergoing dacryocystorhinostomy and showed that chronic dacryocystitis typically presents as chronic nonspecific inflammation with or without fibrosis. Yang and Salour H et al. [30, 31] reported that the most common changes include epithelial necrosis, inflammation, and tissue fibrosis.In our study, the normal lacrimal sac and nasolacrimal duct were lined by two layers of columnar epithelium, consistent with Hirt et al. [18]. In dogs with chronic dacryocystitis, we observed mucosal necrosis, fibrosis, and inflammatory cell infiltration, suggesting close histopathologic similarity between canine and human lacrimal systems. CT dacryocystography has been widely used to diagnose lacrimal diseases in animals such as dogs, cats, and koalas [32–34], as it clearly shows duct anatomy and obstruction sites. Our results indicate that CT, CT 3D reconstruction, and MRI can all localize obstruction, with CT 3D reconstruction providing a simpler and more intuitive visualization of nasolacrimal duct blockage.

In summary, the results of this experimental study demonstrated that nasolacrimal duct obstruction combined with bacterial inoculation could induce lacrimation accompanied by secretions and obstruction of lacrimal irrigation with secretion reflux in dogs. The model established by cauterizing the nasolacrimal duct followed by immediate injection of Staphylococcus aureus exhibited clinical manifestations of dacryocystitis earlier than the model established by injecting Staphylococcus aureus 1 week after nasolacrimal duct cauterization. We analyze that this may be attributed to the repair of the lacrimal duct mucosa 1 week after cauterization, which makes bacterial inoculation difficult to succeed. This may be the reason why the model fails to maintain persistent inflammation, possibly due to the strong regenerative capacity of the canine nasolacrimal duct mucosa, which enables rapid mucosal repair. This rapid healing process limits the persistent existence of inflammatory stimulation, hinders the maintenance of long-term obstruction, and thereby reduces the stability of the chronic dacryocystitis model. In contrast, human chronic dacryocystitis is usually characterized by persistent nasolacrimal duct obstruction, inflammatory cell infiltration, and mucosal fibrosis. This difference may be related to the self-limiting nature of mucosal injury and the transient nature of the inflammatory stimulation adopted in this modeling protocol. To delay the early repair of the mucosa and construct a more stable and reliable chronic dacryocystitis model in subsequent studies, multiple strategies can be considered, including expanding the depth and scope of cauterization, or applying persistent inflammatory stimulation to prolong the duration of mucosal injury and maintain the persistence of inflammation.

We acknowledge that this experiment still has certain limitations. Despite the exploration of cauterization energy and duration, there were a few cases where the canine nasolacrimal duct remained patent or stenotic, which may be due to the fact that the cauterization parameters have not yet reached the optimal range. Although the animals in the experimental group showed obvious symptoms of dacryocystitis, some dogs still had lacrimation without obvious mucopurulent discharge. On the one hand, this may be due to individual differences among dogs; on the other hand, it is necessary to perform multiple bacterial inoculations or further optimize the bacterial concentration. To improve the success rate of chronic dacryocystitis model establishment, more appropriate parameters need to be explored in future studies.

## Conclusion

Our experimental data demonstrate that the anatomical structure of the canine lacrimal duct is similar to that of humans, providing a solid anatomical foundation for modeling chronic dacryocystitis. The method of nasolacrimal duct cauterization combined with Staphylococcus aureus injection can rapidly and effectively induce canine dacryocystitis manifestations while being simple and feasible to perform. Therefore, dogs can be considered as a suitable animal model for further research on chronic dacryocystitis.

This model reliably reproduces key pathological features—including chronic inflammation, ductal obstruction, and bacterial persistence—enabling mechanistic studies and therapeutic evaluation. Its reproducibility and clinical relevance support translational applications in developing novel interventions for human chronic dacryocystitis. If a more persistent chronic inflammatory model is to be constructed, further optimization of surgical parameters and inoculation protocols will still be required.

## Data Availability

The datasets used and/or analysed during the current study are available from the corresponding author on reasonable request.
